# Expression Alterations and Correlative Analysis of TPH1/hsa-miR-194-5p/NEAT1 and MAOA/hsa-miR-1276/NEAT1 Axes in Pediatric Inflammatory Bowel Disease

**DOI:** 10.3390/ijms262411923

**Published:** 2025-12-10

**Authors:** Mehmet Tughan Kiziltug, Mehmet Emin Erdal, Bahar Tasdelen, Ferah Tuncel, Yusuf Usta

**Affiliations:** 1 Department of Pediatrics, Dr. von Hauner Children’s Hospital, University Hospital, Ludwig-Maximilians-Universität München, 80337 Munich, Germany; 2 Department of Medical Biology and Genetics, Faculty of Medicine, Mersin University, Mersin 33110, Türkiye; 3 Department of Biostatistics and Medical Informatics, Faculty of Medicine, Mersin University, Mersin 33110, Türkiye; 4 Department of Medical Pathology, Faculty of Medicine, Mersin University, Mersin 33110, Türkiye; 5 Division for Pediatric Gastroenterology, Hepatology and Nutrition, Department of Pediatrics, Faculty of Medicine, Mersin University, Mersin 33110, Türkiye

**Keywords:** pIBD, serotonin, ncRNAs

## Abstract

Pediatric inflammatory bowel disease (pIBD), comprising ulcerative colitis (UC) and Crohn’s disease (CD), involves complex mechanisms that include non-coding RNAs (ncRNAs), such as microRNAs (miRNAs) and long non-coding RNAs (lncRNAs), alongside enzymes regulating serotonin metabolism. Tryptophan hydroxylase 1 (TPH1) and monoamine oxidase A (MAOA) play critical roles in serotonin turnover and may contribute to intestinal inflammation. We investigated the expression of TPH1, MAOA, hsa-miR-194-5p, hsa-miR-1276, and the lncRNA Nuclear Enriched Abundant Transcript 1 (NEAT1) in intestinal tissue biopsies and peripheral blood from pIBD patients and controls. TPH1 was significantly elevated in the inflamed transverse colon (*p* = 0.034), whereas MAOA was reduced in the ileum (*p* = 0.041) and descending colon (*p* = 0.001), with further decreases in inflamed ileum (*p* < 0.001), ascending (*p* = 0.008), and descending colon (*p* = 0.001). Subgroup analysis revealed decreased MAOA in the ascending colon of UC patients (*p* = 0.011). hsa-miR-194-5p was upregulated in the transverse colon (*p* = 0.015), inflamed transverse (*p* = 0.013) and descending colon (*p* = 0.015), and in blood of UC patients (*p* = 0.01). NEAT1 expression increased in the ascending colon (*p* = 0.042) but decreased in the ileum (*p* = 0.006). Correlation analysis showed strong positive associations between TPH1 and NEAT1 in the ileum (r = 0.945, *p* < 0.01) and transverse colon (r = 0.609, *p* < 0.01). These results highlight region-specific dysregulation of serotonin-related genes and ncRNAs in pIBD, with the TPH1/miR-194-5p/NEAT1 axis potentially contributing to disease pathophysiology and warranting further mechanistic investigation.

## 1. Introduction

Pediatric inflammatory bowel disease, which includes ulcerative colitis and Crohn’s disease, is a chronic and disabling disorder characterized by persistent inflammation of the gastrointestinal tract. The disease presents with recurring episodes that severely impact children’s health and development [1,2,3]. The prevalence of pIBD continues to increase, and nearly one-quarter of all cases are diagnosed before the age of 18 [1]. Pediatric onset introduces unique and often more severe challenges—such as impaired growth, delayed puberty, adolescent psychological challenges, and body image concerns—that are often more complex and severe than those encountered in adult IBD [4,5]. Although the exact cause of IBD remains unclear, it is generally accepted that the disease arises from an abnormal immune response to environmental triggers, including luminal and microbial antigens, in genetically susceptible individuals [6].

Serotonin, or 5-hydroxytryptamine (5-HT), plays a key role in gastrointestinal function, particularly in regulating motility and inflammation [7]. The gastrointestinal tract contains about 95% of the body’s serotonin [8], most of which is synthesized by enterochromaffin cells—specialized enteroendocrine cells that represent the primary source of intestinal 5-HT [9]. Tryptophan is converted into 5-HT through a two-step process involving the enzymes tryptophan hydroxylase (TPH) and aromatic L-amino acid decarboxylase. The rate-limiting enzyme TPH exists in two isoforms: TPH1, expressed in enterochromaffin cells and associated with intestinal inflammation, and TPH2, present in enteric and brain neurons, which regulates motility and neurogenesis [10,11]. Once released, 5-HT is taken up by mucosal enterocytes through the serotonin transporter (SERT) and degraded by monoamine oxidase A, a mitochondrial enzyme responsible for deaminating multiple biogenic amines [10,12].

Altered 5-HT signaling has been observed in both ulcerative colitis and Crohn’s disease, as well as in experimental colitis models [13,14,15,16,17]. Accumulating evidence suggests that disruptions in serotonin synthesis, reuptake, or receptor signaling can substantially influence intestinal inflammation and its complications [14]. Changes in serotonin levels and signaling pathways have been consistently associated with disease activity, indicating that serotonin is a key mediator in both the development and progression of IBD. Nevertheless, more research is needed to fully elucidate the mechanisms by which serotonin affects IBD and to explore potential therapeutic interventions targeting serotonin pathways.

**Figure 1:**
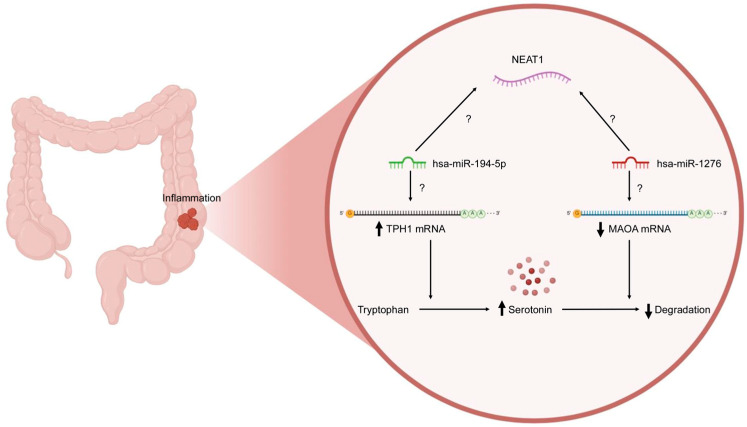
Schematic representation of the role of the TPH1/hsa-miR-194-5p/NEAT1 and MAOA/hsa-miR-1276/NEAT1 axes in the context of pediatric inflammatory bowel disease. The diagram illustrates how NEAT1 interacts with hsa-miR-194-5p and hsa-miR-1276, influencing the expression of TPH1 mRNA (↑ upregulated) and MAOA mRNA (↓ downregulated). TPH1 mRNA facilitates the conversion of tryptophan to serotonin, while the suppression of MAOA mRNA reduces serotonin degradation, leading to increased serotonin levels. Intestinal inflammation is shown to trigger this regulatory mechanism. The extent to which this inflammation-associated disruption of serotonin homeostasis is attributable to the TPH1/hsa-miR-194-5p/NEAT1 and MAOA/hsa-miR-1276/NEAT1 axes remains to be determined. Created with Biorender.com.

Noncoding RNAs are derived from the genome’s regions that do not code for proteins but instead produce transcripts that regulate gene expression and protein function. The two major classes—microRNAs and long noncoding RNAs—play essential roles in modulating diverse biological processes [18]. Since ncRNAs are crucial for regulating biological processes across various cell types and tissues, their dysregulation has become increasingly associated with the development and progression of numerous diseases, including neurological [19], cardiovascular [20], cancer [21] and inflammatory diseases [22]. For example, tumor necrosis factor-alpha (TNFα), a well-established activator of the NF-κB signaling pathway, plays a critical role in exacerbating inflammation, and its stimulation significantly downregulated the expression of miR-1276, suggesting a potential regulatory mechanism in inflammatory processes [23]. miR-194 inhibits the inflammatory Toll-like receptor 4 (TLR4) pathway by targeting tumor necrosis factor receptor-associated factor 6 (TRAF6) [24]. The lncRNA Neat1 promotes inflammasome activation by interacting with NLRP3, NLRC4, and AIM2 in macrophages, enhancing their assembly and pro-caspase-1 processing, and thereby acting as a common mediator of innate immunity [25]. Despite extensive research on individual ncRNAs, the interplay among mRNA, miRNA, and lncRNA components in the serotonergic pathway of pIBD remains largely unexplored. Based on in silico predictions, we selected hsa-miR-194-5p and hsa-miR-1276, which showed the highest binding affinity to TPH1 and MAOA, respectively, and have reported roles in inflammatory and serotonergic regulation. Given the limited pediatric data and the lack of studies examining NEAT1 in this context, these two miRNAs were prioritized to investigate their potential involvement in serotonin-related dysregulation in pIBD. We therefore hypothesized that the TPH1/hsa-miR-194-5p/NEAT1 and MAOA/hsa-miR-1276/NEAT1 axes may contribute to serotonin-driven inflammation in pIBD (Figure 1).

To test this, we conducted molecular analyses aimed at elucidating how miR-194-5p and miR-1276, together with NEAT1, modulate TPH1- and MAOA-associated serotonergic signaling in the pathogenesis of pIBD. By identifying specific interactions between these ncRNAs and key inflammatory mediators, we sought to uncover potential therapeutic targets for future intervention strategies in pediatric IBD.

## 2. Results

### 2.1. Study Cohort

A total of 44 mucosal biopsies were obtained from 22 pediatric patients with inflammatory bowel disease, comprising 9 with ulcerative colitis, 10 with Crohn’s disease, and 3 with indeterminate colitis (IC). 22 age- and sex-matched healthy controls were also included; these individuals underwent endoscopy due to clinical suspicion of intestinal disease but were confirmed to be disease-free. The mean age of patients with IBD was 14.45 ± 2.75 years, with 45.5% female, whereas the mean age of healthy controls was 13.09 ± 4.13 years, with 40.9% female (Table 1). No significant differences in age or sex were observed between the groups.

### 2.2. Prediction and Construction of TPH1/hsa-miR-194-5p/NEAT1 and MAOA/hsa-miR-1276/NEAT1 Axes

NEAT1 was selected from the LncRNADisease v3.0 database [26] for its reported association with inflammatory bowel disease (Figure S1). To investigate the potential interactions among lncRNAs and mRNAs, we developed a lncRNA–miRNA–mRNA network. We utilized widely-used miRNA target prediction tools to forecast interactions between various lncRNAs and miRNAs. Following this, we predicted miRNA binding sites and target mRNAs using proprietary software informed by miRDB v6.0 bioinformatics tool [27,28]. miRNAs interacting with NEAT1 were selected for their high targeting scores for TPH1 and MAOA, as assessed using the ENCORI/starBase v2.0 bioinformatics tool [29]. ENCORI analysis identified two 7mer-m8 binding sites for hsa-miR-194-5p within NEAT1, and one 7mer-m8 site together with two 8mer seed-match sites for hsa-miR-1276, supporting the biological plausibility of the proposed TPH1/miR-194-5p/NEAT1 and MAOA/miR-1276/NEAT1 regulatory axes. The final lncRNA–miRNA–mRNA interaction network was constructed using Cytoscape (v3.10.2) [30], integrating data on lncRNAs, miRNAs, and mRNAs (Figure S2). Among these, hsa-miR-194-5p was identified as a high-confidence miRNA for targeting TPH1, while hsa-miR-1276 was identified as a high-confidence miRNA for targeting MAOA.

### 2.3. Functional Annotations of TPH1, MAOA, and NEAT1

Gene ontology analysis was conducted to elucidate the biological functions of TPH1, MAOA, and NEAT1 in serotonin metabolism. The results are illustrated in Figure S3, which demonstrate the associations of TPH1, MAOA, and NEAT1 with gene ontology categories, specifically emphasizing their respective biological processes, molecular functions, and wikipathway analysis, thereby highlighting the roles these genes play in the pathophysiology of pIBD.

### 2.4. mRNA Expression Analysis of TPH1 and MAOA

**Figure 2:**
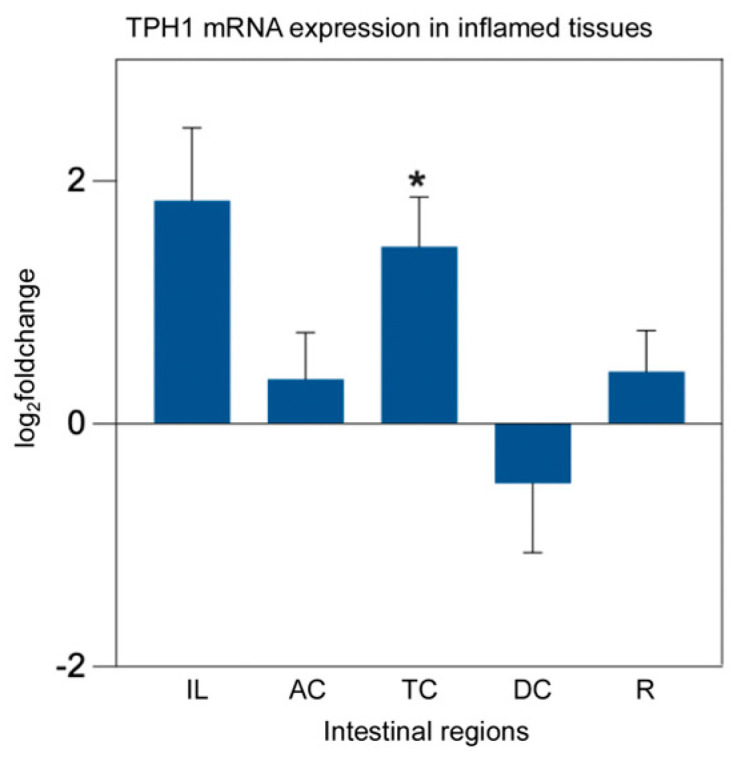
TPH1 mRNA expression analysis in inflamed intestinal tissues from pIBD patients compared to healthy controls. TPH1 expression was significantly higher in the inflamed transverse colon compared to controls (independent *t*-test, *p* = 0.034; df = 41; control *n* = 22, inflamed *n* = 21; log_2_FC = 1.46 ± 0.41 SEM), whereas no significant differences were observed in other intestinal regions (*p* > 0.05). All data are presented as log_2_(fold change) ± SEM (* *p* < 0.05). Ileum (IL), ascending colon (AC), transverse colon (TC), descending colon (DC), and rectum (R).

In patients with Crohn’s disease, TPH1 expression in the colon was significantly elevated in inflamed regions compared to noninflamed regions and healthy controls [31]. However, a meta-analysis of the transcriptome revealed that TPH1 expression was significantly reduced in inflamed colonic biopsies from patients with UC, but not in those with CD [12]. To investigate these differential expressions in pIBD patients, we measured mRNA expression levels in biopsies from the ileum (IL), ascending colon (AC), transverse colon (TC), descending colon (DC), and rectum (R), categorized by control-pIBD, control-inflamed, and control-UC-CD-IC groups. There was no significant change in TPH1 expression between the control-pIBD and control-UC-CD-IC groups (*p* > 0.05), whereas TPH1 expression was significantly higher in the inflamed transverse colon compared to healthy controls (*p* = 0.034) (Figure 2).

**Figure 3:**
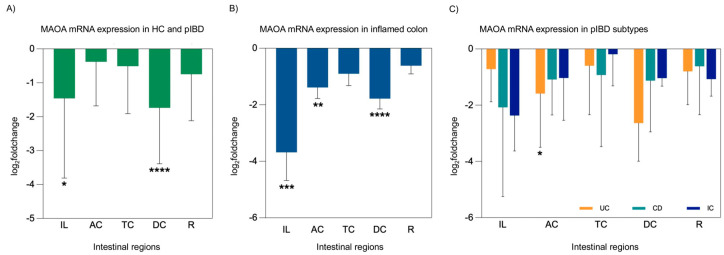
MAOA mRNA expression analysis in intestinal tissues from pIBD patients compared to healthy controls, including overall patient comparisons, inflamed tissue analyses, and disease subtype evaluations (UC, CD, and IC). MAOA expression was decreased in all intestinal regions of pIBD patients compared to controls, with the most pronounced reductions observed in ileum and the descending colon (independent *t*-test, *p* = 0.041 and 0.001; df = 42; control *n* = 22, patient *n* = 22; log_2_FC = −1.46 ± 0.66 SEM and −1.74 ± 0.48 SEM, respectively) (**A**). Inflamed tissues showed a stronger reduction, particularly in the ileum, ascending colon, and descending colon (independent *t*-test, *p* < 0.001, *p* = 0.008, and 0.001; df = 32, 40, and 41; control *n* = 22, inflamed *n* = 12, 20, and 21; log_2_FC = −3.68 ± 1.00, −1.39 ± 0.39, and −1.78 ± 0.37 SEM, respectively) (**B**). A significant decrease in MAOA expression was observed specifically in patients with UC in the AC region (one-way ANOVA, Bonferroni post hoc test, *p* = 0.011; df = 29; control *n* = 22, UC *n* = 9; log_2_FC = −1.59 ± 0.64 SEM) (**C**). All data are presented as log_2_(fold change) ± SEM (* *p* < 0.05, ** *p* < 0.01, *** *p* < 0.001, and **** *p* < 0.0001). Ileum (IL), ascending colon (AC), transverse colon (TC), descending colon (DC), rectum (R), ulcerative colitis (UC), Crohn’s disease (CD), and indeterminate colitis (IC).

MAO activity was found to be decreased by 34% in patients experiencing the acute stage of irritable bowel syndrome, whereas it was increased by 44% in patients with nonspecific ulcerative colitis [32]. Biogenic amines like histamine and serotonin, recognized as key inflammatory mediators, are predominantly oxidized by MAOA. Reducing these pro-inflammatory mediators at inflammation sites could potentially convert naive monocytes from a pro-inflammatory to an anti-inflammatory state, thereby alleviating inflammatory symptoms [33]. To evaluate the role of MAOA as a contributor to IBD pathogenesis in intestinal tissues, MAOA expression analysis was performed in five different intestinal regions. Our analysis demonstrated a significant reduction in MAOA mRNA expression levels in the ileum (*p* = 0.041) and descending colon (*p* = 0.001) of pIBD patients. Additionally, MAOA expression was significantly reduced in the inflamed ileum (*p* < 0.001), inflamed ascending colon (*p* = 0.008), and inflamed descending colon (*p* = 0.001). When assessing MAOA expression across pIBD subtypes, a significant decrease was observed in the ascending colon region of patients with UC (*p* = 0.011) (Figure 3).

### 2.5. miRNA Expression Analysis of hsa-miR-194-5p and hsa-miR-1276

**Figure 4:**
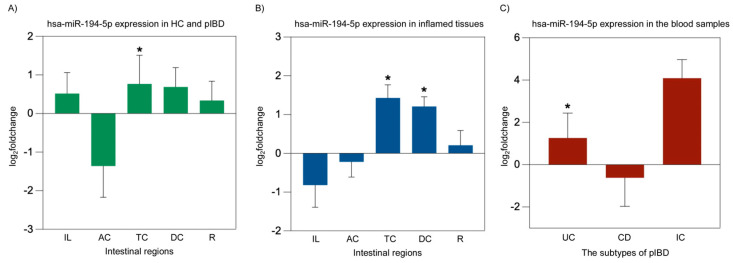
hsa-miR-194-5p miRNA expression analysis in overall patient intestinal tissues, inflamed intestinal tissues, and blood from pIBD patients compared to healthy controls. A significant increase in hsa-miR-194-5p expression was observed in the transverse colon of pIBD patients compared to healthy controls (independent *t*-test, *p* = 0.015; df = 42; control *n* = 22, patient *n* = 22; log_2_FC = 0.77 ± 0.74 SEM) (**A**). Inflamed tissues showed significant upregulation of hsa-miR-194-5p in the transverse and descending colon (independent *t*-test, *p* = 0.013 and *p* = 0.015; df = 41 and 41; control *n* = 22, inflamed *n* = 21 and 21; log_2_FC = 1.43 ± 0.34 and 1.21 ± 0.25 SEM, respectively) (**B**). In blood samples, a significant upregulation of hsa-miR-194-5p expression was detected in UC patients compared to healthy controls (one-way ANOVA, Bonferroni post hoc test, *p* = 0.01; df = 29; control *n* = 22, UC *n* = 9; log_2_FC = 1.26 ± 1.18 SEM) (**C**). All data are presented as log_2_(fold change) ± SEM (* *p* < 0.05). Ileum (IL), ascending colon (AC), transverse colon (TC), descending colon (DC), rectum (R), ulcerative colitis (UC), Crohn’s disease (CD), and indeterminate colitis (IC).

We evaluated whether the microRNAs hsa-miR-194-5p and hsa-miR-1276 may play roles in the pathogenesis of IBD. hsa-miR-194-5p expression was significantly increased in TC from IBD patients (*p* = 0.015). Additionally, hsa-miR-194-5p levels were higher in inflamed-TC (*p* = 0.013) and inflamed-DC (*p* = 0.015). Furthermore, a significant upregulation of hsa-miR-194-5p was also observed in the blood of patients with UC (*p* = 0.01) (Figure 4). In contrast, no significant differences were observed in the expression levels of hsa-miR-1276 in any of the studied conditions (*p* > 0.05). The endogenous control hsa-miR-26b-5p exhibited stable Ct values across all intestinal segments, blood samples, inflammatory states, and patient diagnostic subgroups, with no significant differences between control and patient samples, between inflamed and non-inflamed patient tissues, or among UC, CD, and IC groups (all *p* > 0.05), supporting its suitability as a reference miRNA for normalization.

### 2.6. lncRNA Expression Analysis of NEAT1

**Figure 5:**
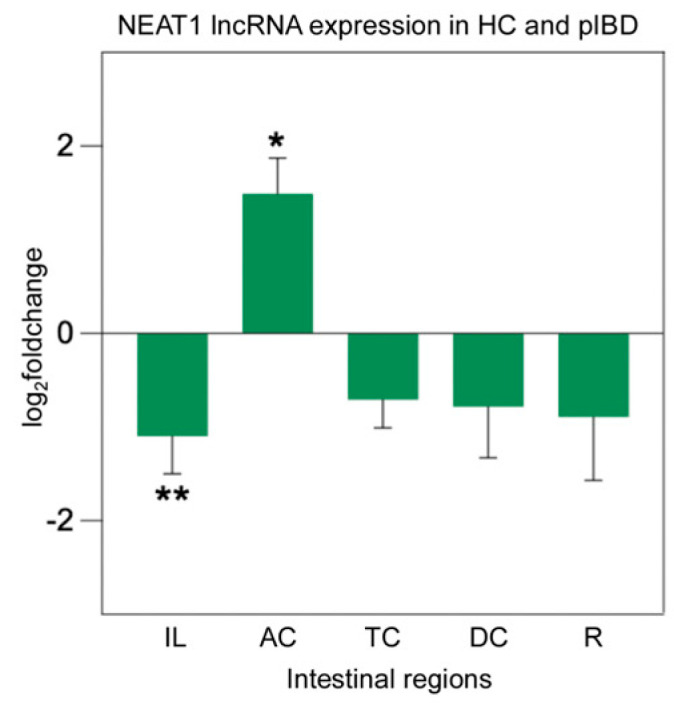
NEAT1 lncRNA expression analysis in overall patient intestinal tissues from pIBD patients compared to healthy controls. A significant decrease in NEAT1 expression was observed in the ileum compared to healthy controls (independent *t*-test, *p* = 0.006; df = 42; control *n* = 22, patient *n* = 22; log_2_FC = −1.10 ± 0.40 SEM). In contrast, a significant increase in NEAT1 expression was detected in the ascending colon (independent *t*-test, *p* = 0.042; df = 42; control *n* = 22, patient *n* = 22; log_2_FC = 1.49 ± 0.38 SEM). All data are presented as log_2_(fold change) ± SEM (* *p* < 0.05 and ** *p* < 0.01). Ileum (IL), ascending colon (AC), transverse colon (TC), descending colon (DC), and rectum (R).

Previous studies have shown that NEAT1 expression is significantly elevated in both IBD patient-derived cells and animal models of the disease by promoting inflammation [34,35]. To assess whether NEAT1 is involved in the development of IBD, NEAT1 expression was quantified using qPCR. In patients with IBD, the analysis indicated a notable decrease in the ileum (*p* = 0.006) and a significant increase in NEAT1 expression in AC (*p* = 0.042) (Figure 5).

### 2.7. Correlation Analysis of TPH1/hsa-miR-194-5p/NEAT1 and MAOA/hsa-miR-1276/NEAT1 in IBD Patients

**Figure 6:**
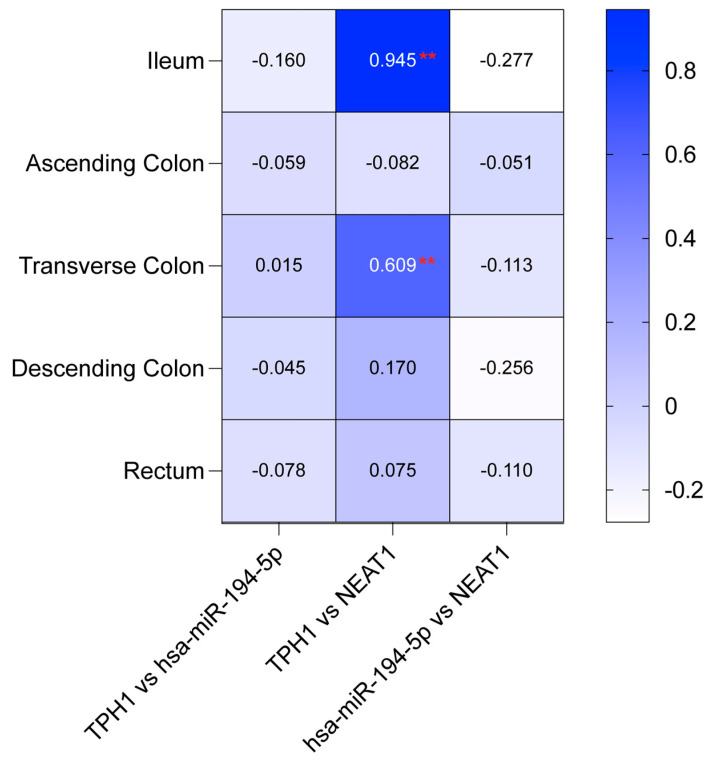
Correlation coefficients for the TPH1/hsa-miR-194-5p/NEAT1 axis across intestinal regions. Heatmap showing Pearson correlation coefficients between TPH1, hsa-miR-194-5p, and NEAT1 expression in five intestinal regions: ileum, ascending colon, transverse colon, descending colon, and rectum (Pearson correlation analysis, df = 42; control *n* = 22, patient *n* = 22). Strong positive correlations are observed between TPH1 and NEAT1 in the ileum and transverse colon (** *p* < 0.01), while other pairwise comparisons show no significant correlations.

Figure 6 indicates a robust positive correlation between TPH1 and NEAT1 in the ileum (r = 0.945, *p* < 0.01) and the transverse colon (r = 0.609, *p* < 0.01). Conversely, the relationships between TPH1 and hsa-miR-194-5p, as well as NEAT1 and hsa-miR-194-5p, were generally weak or negative across all intestinal regions (Figure 6). These findings underscore a strong link between TPH1 and NEAT1, while interactions with hsa-miR-194-5p vary across different segments of the colon. To assess potential regulatory interactions, we analyzed the expression relationships among MAOA, hsa-miR-1276, and NEAT1; however, no significant correlations were observed (*p* > 0.05).

## 3. Discussion

Pediatric inflammatory bowel disease is a long-term and severe condition that typically shows a more aggressive progression and broader gastrointestinal impact than inflammatory bowel disease that begins in adulthood. [5]. Although pIBD is regarded as a chronic idiopathic disorder, its development is considered to result from multiple interacting factors, including genetic predispositions, environmental factors, the microbiome, and the immune system [4,36]. Recent research has also emphasized the importance of the brain–gut axis—a bidirectional communication network in which serotonin acts as a key signaling molecule connecting the enteric and central nervous systems [37,38,39]. Dysregulated serotonin signaling is particularly relevant to IBD, where elevated 5-HT levels are frequently observed. This imbalance may contribute to intestinal inflammation, as increased serotonin availability has been linked to enhanced inflammatory activity in both human and experimental studies [15,40,41,42]. Therefore, alterations in serotonin metabolism and signaling likely play a central role in the pathogenesis of pIBD, influencing disease progression and clinical manifestation.

In this study, we analyzed the mRNA expression of TPH1 and MAOA across distinct intestinal regions, as these genes are essential for serotonin synthesis and degradation, respectively [43]. We also examined the expression of hsa-miR-194-5p and hsa-miR-1276—miRNAs that target TPH1 and MAOA—as well as the lncRNA NEAT1, which may modulate these genes through miRNA sponging. This integrated approach aimed to clarify whether dysregulation within this network contributes to altered serotonin biosynthesis and degradation in pIBD.

Perturbations in serotonin metabolism have long been implicated in the development of IBD. Experimental models have shown that pharmacological suppression of 5-HT production alleviates inflammation [44,45]. Similarly, genetic deletion of *Tph1* significantly reduces the severity of colitis, indicating that 5-HT exacerbates intestinal inflammation [15]. Haq et al. (2021) has shown that lower intestinal 5-HT levels enhance autophagy and reduce inflammation, while restoring serotonin impairs autophagy and worsens inflammatory responses [41]. These findings are consistent with reports of elevated TPH1 expression in the colonic mucosa of Crohn’s disease patients, suggesting that TPH1 activity is closely linked to inflammatory processes [14,46]. Conversely, a meta-analysis of the transcriptome revealed significantly reduced TPH1 expression in inflamed colonic biopsies from UC patients, but not in CD patients, suggesting a more pronounced inhibition of serotonin synthesis in UC patients with active inflammation [12]. Analysis of TPH1 indicated that its expression remained stable, showing no significant changes during either the active or inactive phases of IBD [47]. In our study, TPH1 expression did not differ significantly between control and disease groups overall, but it was notably higher in the inflamed transverse colon compared to healthy tissue. This observation suggests that TPH1 upregulation may be associated with active inflammation, reinforcing its potential as a marker of inflammatory activity.

MAOA, the primary enzyme responsible for serotonin degradation, is critical for maintaining homeostasis in intestinal serotonin signaling [10]. Meta-analyses have shown that patients with active IBD reduced MAO expression, indicating inhibited degradation in their intestinal tissues [12]. The decrease in MAOA levels may reflect altered macrophage activation, leading to impaired resolution of inflammation and a shift toward a proinflammatory state [33]. In our data, MAOA mRNA expression was significantly reduced in the ileum and descending colon, particularly in inflamed regions such as the ileum, ascending colon, and descending colon. This pattern aligns with previous reports of downregulated MAOA in UC and supports the concept that decreased serotonin catabolism contributes to the persistence of inflammation. The regional variation in MAOA expression highlights its potential as both a mechanistic and therapeutic target in IBD.

Recent research has identified a strong association between lncRNA NEAT1 and the pathogenesis of IBD [48,49,50,51]. Elevated NEAT1 expression in colitis mouse models has been shown to exacerbate inflammation by disrupting epithelial barrier integrity and modulating macrophage polarization through exosome-mediated signaling. High NEAT1 levels promote M1 macrophage activation and inflammatory cytokine release, whereas NEAT1 inhibition restores barrier function and reduces inflammation [34]. Pan et al. (2021) further elucidated NEAT1’s involvement in driving intestinal inflammation by regulating TNFRSF1B and activating the NF-κB pathway, which results in heightened levels of inflammatory cytokines and promotes NF-κB p65 translocation into the nucleus [35]. NEAT1 knockdown effectively reduced these inflammatory markers, though elevated TNFRSF1B could counteract this effect [35]. In ulcerative colitis, NEAT1 upregulation contributes to inflammation and epithelial injury through multiple regulatory pathways. By suppressing miR-603, NEAT1 increases cell viability and reduces apoptosis and cytokine production via the NEAT1/miR-603/FGF9 axis [52]. It also modulates cytokine release and cell survival through the miR-204–5p/PI3K–Akt3 pathway, suggesting potential for therapeutic targeting in colitis [53]. Furthermore, elevated NEAT1 enhances glucose metabolism in UC intestinal epithelial cells by downregulating miR-410–3p, which controls lactate dehydrogenase A (LDHA); NEAT1 silencing restores metabolic balance and epithelial function [54]. In our study, NEAT1 expression was significantly increased in the ascending colon of pIBD cases but decreased in the ileum. This dual pattern is consistent with NEAT1’s dynamic involvement in inflammatory regulation, suggesting that its expression may vary depending on tissue context or disease stage. Elevated NEAT1 levels likely contribute to inflammation through barrier dysfunction and macrophage polarization, whereas reduced expression may represent a compensatory or regulatory response.

NEAT1 regulates IBD pathogenesis by acting as a competitive endogenous RNA (ceRNA) that modulates miRNA expression [34,35,52,53,54]. The regulatory interplay among NEAT1, miRNAs, and serotonergic genes provides new insight into the molecular mechanisms of pIBD. Our data revealed that hsa-miR-194-5p was consistently upregulated across inflamed tissues and blood samples, indicating its strong association with pIBD pathogenesis. In contrast, hsa-miR-1276 showed no significant changes, suggesting a more limited role. The observed correlations between TPH1, NEAT1, and hsa-miR-194-5p indicate a potential ceRNA mechanism, where NEAT1 sponges hsa-miR-194-5p to enhance TPH1 expression and serotonin synthesis, thereby amplifying inflammation. This region-specific regulation highlights the complexity of ncRNA-mediated control in intestinal inflammation.

To our knowledge, this study provides the first integrative analysis of serotonin-associated genes and noncoding RNAs in pIBD. We identify a distinct transcriptional profile characterized by upregulation of TPH1, downregulation of MAOA, and region-specific dysregulation of NEAT1 within intestinal tissues. Unlike prior reports that have largely focused on adult IBD or individual molecular components, our work delineates—for the first time—the coordinated regulation of the TPH1/miR-194-5p/NEAT1 and MAOA/miR-1276/NEAT1 axes in the pediatric context, highlighting the contribution of disrupted serotonergic signaling to intestinal inflammation. Moreover, by examining both control–patient and control–inflamed tissue comparisons across multiple intestinal regions, this study reveals that transcriptional variation in pIBD is influenced by the anatomical localization of disease activity. Although limited by cohort size, these findings establish an important proof-of-concept for region-specific molecular dysregulation in early-onset IBD and provide a conceptual framework for future transcriptomic and spatial analyses aimed at refining the molecular taxonomy of pediatric intestinal inflammation.

Despite revealing significant expression changes and correlations among serotonin-related genes and non-coding RNAs, this study is limited by its descriptive nature. Expression analyses alone cannot confirm the causal or mechanistic involvement of these molecules in pIBD pathogenesis. Functional validation through in vitro and in vivo studies, such as gene silencing, overexpression, and reporter assays, will be required to determine whether the observed dysregulations actively contribute to inflammatory pathways or reflect secondary effects of tissue inflammation. Furthermore, the modest sample size and lack of protein-level or cellular localization data restrict broader generalization of the results. Future studies integrating transcriptomic, proteomic, and functional assays will be essential to establish the mechanistic relevance of the TPH1/miR-194-5p/NEAT1 and MAOA/miR-1276/NEAT1 axes in pIBD.

## 4. Materials and Methods

### 4.1. Study Population

This study was conducted in compliance with all relevant ethical standards and received approval from the Clinical Research Ethics Committee of Mersin University, Mersin, Türkiye (Approval No: 2019/12). The research adhered to the principles of the Declaration of Helsinki (2013).

The inclusion criteria for children with suspected pIBD were: (1) age between 6 and 18 years; (2) clinical symptoms such as abdominal pain, diarrhea, rectal bleeding, or weight loss persisting for more than 1 month, or perianal disease, anemia, malnutrition, arthritis, and similar findings lasting more than 6 months; and (3) newly diagnosed pIBD. Exclusion criteria were: (1) age below 5 year or above 18 years; (2) very early-onset pIBD; and (3) recurrent pIBD.

A total of 220 mucosal biopsies were collected, including samples from 22 pediatric patients with inflammatory bowel disease (9 with ulcerative colitis, 10 with Crohn’s disease, and 3 with indeterminate colitis) and 22 healthy controls who underwent endoscopy due to suspected intestinal disease but were found to be disease-negative. Among the pIBD patients, pathologically confirmed inflamed tissue samples were obtained from the ileum (*n* = 12), ascending colon (*n* = 20), transverse colon (*n* = 21), descending colon (*n* = 21), and rectum (*n* = 22).

To minimize potential confounding arising from physiological regional variation in gene expression, inflamed and control biopsies were obtained from matching intestinal regions (e.g., ileum vs. ileum, transverse colon vs. transverse colon). This design ensured that all analyses reflected disease-associated transcriptional alterations, independent of baseline anatomical variability.

All patients and controls included in this study were treatment-naïve and presented to the clinic for the first time with suspected inflammatory bowel disease. Individuals with prior diagnosis, ongoing medical follow-up, or any history of pharmacological therapy—including corticosteroids, biologics, immunomodulators, or selective serotonin reuptake inhibitors (SSRIs)—were excluded to eliminate potential confounding effects on serotonin pathway gene expression. Peripheral blood samples were collected immediately before anesthesia induction, and all mucosal biopsies were obtained under anesthesia during diagnostic endoscopy. This ensured that gene expression data reflected the untreated baseline state of the disease, minimizing bias related to therapeutic interventions.

Pediatric inflammatory bowel disease was diagnosed according to the internationally accepted revised Porto criteria established by the European Society for Paediatric Gastroenterology, Hepatology, and Nutrition (ESPGHAN) [55]. These criteria combine clinical presentation, endoscopic findings, and histopathological evaluation to ensure a comprehensive and standardized diagnosis. Histopathological assessment of intestinal biopsies was performed by an experienced gastrointestinal pathologist, with particular attention to architectural distortion, crypt abscess formation, epithelial damage, granuloma presence, and inflammatory cell infiltration within the lamina propria. Biopsies exhibiting these unequivocal pathological features were classified as inflamed, whereas those with preserved architecture and absence of active inflammatory lesions were categorized as non-inflamed. This approach ensured consistent characterization of tissue inflammation across patients and intestinal segments, in line with internationally recognized diagnostic standards for pIBD.

Written informed consent was obtained from all participants or their guardians prior to sample collection, ensuring adherence to ethical standards and participant comprehension.

### 4.2. In Silico Prediction and Construction of TPH1/miR-194-5p/NEAT1 and MAOA/miR-1276/NEAT1 Regulatory Networks with Gene Ontology Analysis

NEAT1 was selected from the LncRNADisease v3.0 database due to its reported association with inflammatory bowel disease. Potential interactions among lncRNAs, miRNAs, and mRNAs were predicted using miRDB v6.0 and ENCORI/starBase v2.0. Candidate miRNAs with high targeting scores for TPH1 and MAOA were identified, among which hsa-miR-194-5p and hsa-miR-1276 were selected. The lncRNA–miRNA–mRNA regulatory network was constructed and visualized using Cytoscape v3.10.2 (Cytoscape Consortium, San Diego, CA, USA). Additionally, Gene Ontology (GO) and Kyoto Encyclopedia of Genes and Genomes (KEGG) pathway analyses of lncRNA target genes and mRNAs were conducted using the SRplot (NewCore Biotech, Shanghai, China) online platform to elucidate the associated biological processes, cellular components, molecular functions, and signaling pathways [56].

### 4.3. Sample Collection and RNA Extraction

Peripheral blood samples were collected preoperatively into ethylenediaminetetraacetic acid (EDTA)-containing tubes. Tissue lysates were homogenized in TRIzol reagent (Thermo Fisher Scientific, Waltham, MA, USA) by repeated aspiration and dispensing using a 23-gauge syringe and needle. Total RNA was extracted from whole blood and biopsy samples by using TRIzol [57]. To purify lncRNA, RNA was treated with DNase I (Thermo Fisher Scientific, Waltham, MA, USA) to remove DNA, precipitated with 10M LiCl (Merck, Darmstadt, Germany), and washed by 70% ethanol (Merck, Darmstadt, Germany) [58,59].

### 4.4. Reverse Transcription-Quantitative Polymerase Chain Reaction (RT-qPCR)

cDNA synthesis for mRNA, miRNA, and lncRNA was performed by RevertAid First Strand cDNA Synthesis Kit (Thermo Fisher Scientific, Waltham, MA, USA) as described previously [60,61]. ACTB was used as the endogenous control for mRNAs and lncRNAs, while hsa-miR-26b-5p served as the internal control for miRNAs. Real-time PCR analysis was conducted by TaqMan™ Universal PCR-Mastermix (Applied Biosystems, Foster City, CA, USA) following the manufacturer’s protocol.

All qPCR reactions were normalized to Applied Biosystems™ Total RNA Control (Human) Applied Biosystems, Foster City, CA, USA, which served as the universal technical calibrator. The expression levels of target transcripts were quantified using the comparative 2^−ΔΔCt^ method [62] on an Applied Biosystems™ 7500 Real-Time PCR System (Applied Biosystems, Foster City, CA, USA) and expressed as log_2_(fold-change) values. Statistical comparisons were subsequently performed between experimental groups based on these normalized and log-transformed data.

The sequences of primers and probes are listed in Table S1.

### 4.5. Statistical Analysis

Statistical analyses were conducted using Statistica 11 (StatSoft Inc., Tulsa, OK, USA), and graphical representations were generated with GraphPad Prism 10.4.2 (GraphPad Software, San Diego, CA, USA). Data normality was assessed with the Shapiro–Wilk test. Expression levels of mRNAs, miRNAs, and lncRNAs were log-transformed to satisfy normality assumptions; group comparisons between patients and controls, as well as between inflamed tissues and controls, were performed using the independent *t* test, with *p*-values adjusted for multiple comparisons via the Benjamini–Hochberg procedure. For datasets meeting normality assumptions with three or more groups, differences were evaluated using one-way ANOVA followed by the Bonferroni post hoc test. Relationships between continuous variables were examined using Pearson’s correlation analysis, and statistical significance was defined as *p* < 0.05. In addition, to evaluate the stability of the endogenous control miR-26b-5p, Ct values were compared between control and patient samples for each intestinal segment and in blood, between control and inflamed tissues, and among diagnostic subgroups (Control, UC, CD, IC). Non-normal datasets were analyzed using Mann–Whitney U tests, normally distributed data using unpaired *t* tests, and comparisons involving more than two independent groups using the nonparametric Kruskal–Wallis test, with *p* < 0.05 considered statistically significant.

## 5. Conclusions

This study underscores the pivotal role of serotonin metabolism in pediatric inflammatory bowel disease pathogenesis, with elevated TPH1 expression in the inflamed transverse colon and reduced MAOA expression in regions like the ileum and descending colon driving an inflammatory imbalance. The lncRNA NEAT1, upregulated in the ascending colon, likely amplifies serotonin synthesis by acting as a ceRNA, particularly through its strong correlations with TPH1 in the ileum (r = 0.945, *p* < 0.01) and transverse colon (r = 0.609, *p* < 0.01). The weak correlations between TPH1 and hsa-miR-194-5p (e.g., r = -0.160 in the ileum) suggest NEAT1 sponges this miRNA, enhancing TPH1 expression and inflammation. The negligible role of hsa-miR-1276 highlights the selective regulatory function of hsa-miR-194-5p in pIBD. These molecular interactions position NEAT1 and hsa-miR-194-5p as promising non-invasive biomarkers for pIBD diagnosis and disease activity monitoring, offering enhanced patient compliance through repeatable, non-invasive sampling compared to conventional diagnostic approaches. This approach facilitates timely detection and tracking of disease fluctuations, critical for managing pIBD’s dynamic progression. A comprehensive transcriptomic analysis integrating RNA-seq, small RNA-seq, and miRNA target prediction with functional validation (e.g., NEAT1 knockdown) is recommended to further elucidate mRNA-miRNA-lncRNA networks, offering insights into pIBD pathogenesis and novel treatment strategies.

## Figures and Tables

**Table 1:** Outline of the demographic information for both healthy controls and patients diagnosed with pIBD.

	Healthy Controls	Patients with pIBD
Participants	22	22
Mean age in years	13.09 ± 4.13	14.45 ± 2.75
% Female	40.9	45.5

## Data Availability

Data supporting the conclusions of this study can be obtained from the corresponding author, contingent upon appropriate requests and adherence to ethical guidelines.

## Supplementary Materials

The following supporting information can be downloaded at: https://www.mdpi.com/article/10.3390/ijms262411923/s1.

## Abbreviations

The following abbreviations are used in this manuscript:

pIBD	Pediatric inflammatory bowel disease
UC	Ulcerative colitis
CD	Crohn’s disease
IC	Indeterminate colitis
ncRNAs	Non-coding RNAs
miRNAs	MicroRNAs
lncRNAs	Long non-coding RNAs
TPH	Tryptophan hydroxylase
MAOA	Monoamine oxidase A
NEAT1	Nuclear Enriched Abundant Transcript 1
5-HT	5-hydroxytryptamine
TNFα	Tumor necrosis factor-alpha
TLR4	Toll-like receptor 4
TRAF6	Tumor necrosis factor receptor-associated factor 6
IL	Ileum
AC	Ascending colon
TC	Transverse colon
DC	Descending colon
R	Rectum
