# Supplementary material for: Expression Alterations and Correlative Analysis of TPH1/hsa-miR-194-5p/NEAT1 and MAOA/hsa-miR-1276/NEAT1 Axes in Pediatric Inflammatory Bowel Disease
Source: Int J Mol Sci. 2025 Dec 10;26(24):11923. doi: 10.3390/ijms262411923 (PMC12732696; doi:10.3390/ijms262411923)
Supplement: Supplementary file 1 [file ijms-26-11923-s001.zip › ijms-3883434-supplementary.pdf]

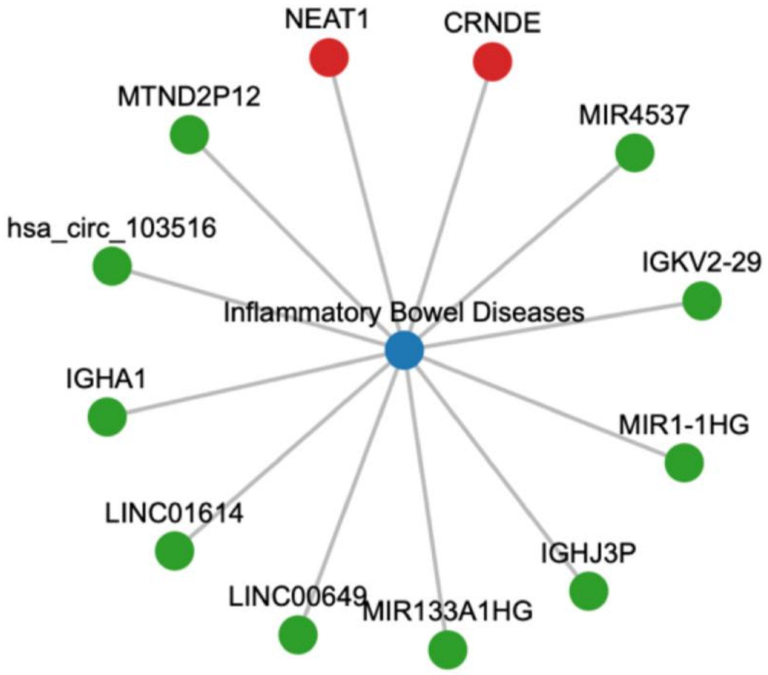

Figure S1. Interaction network illustrating the associations between pIBD and lncRNAs. Red: Top causal ncRNAs for IBD, green: Unknown causal ncRNAs for IBD.

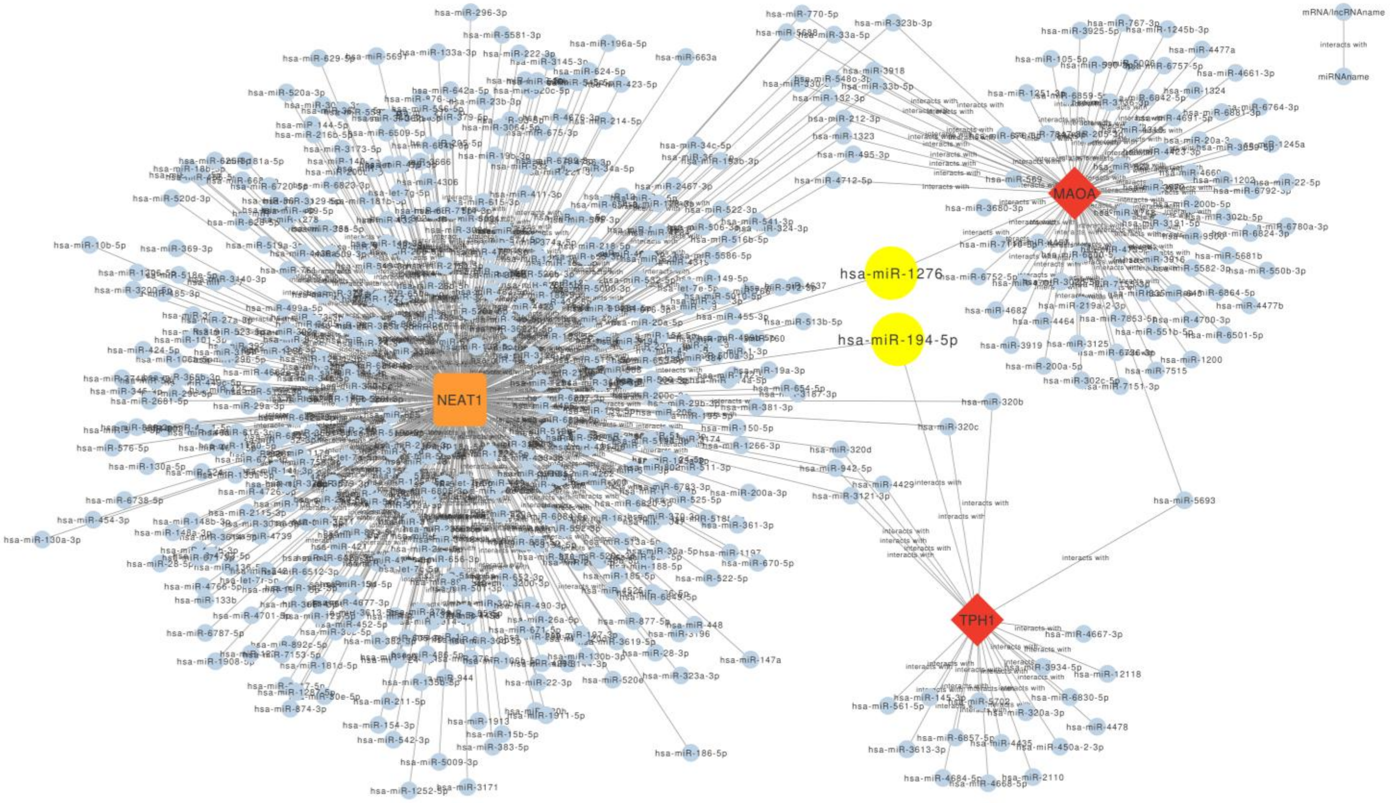

Figure S2. In the network representation, a lncRNA is depicted as orange square, mRNAs as red rhombuses, and miRNAs as green circles, with target miRNAs shown as yellow circles.

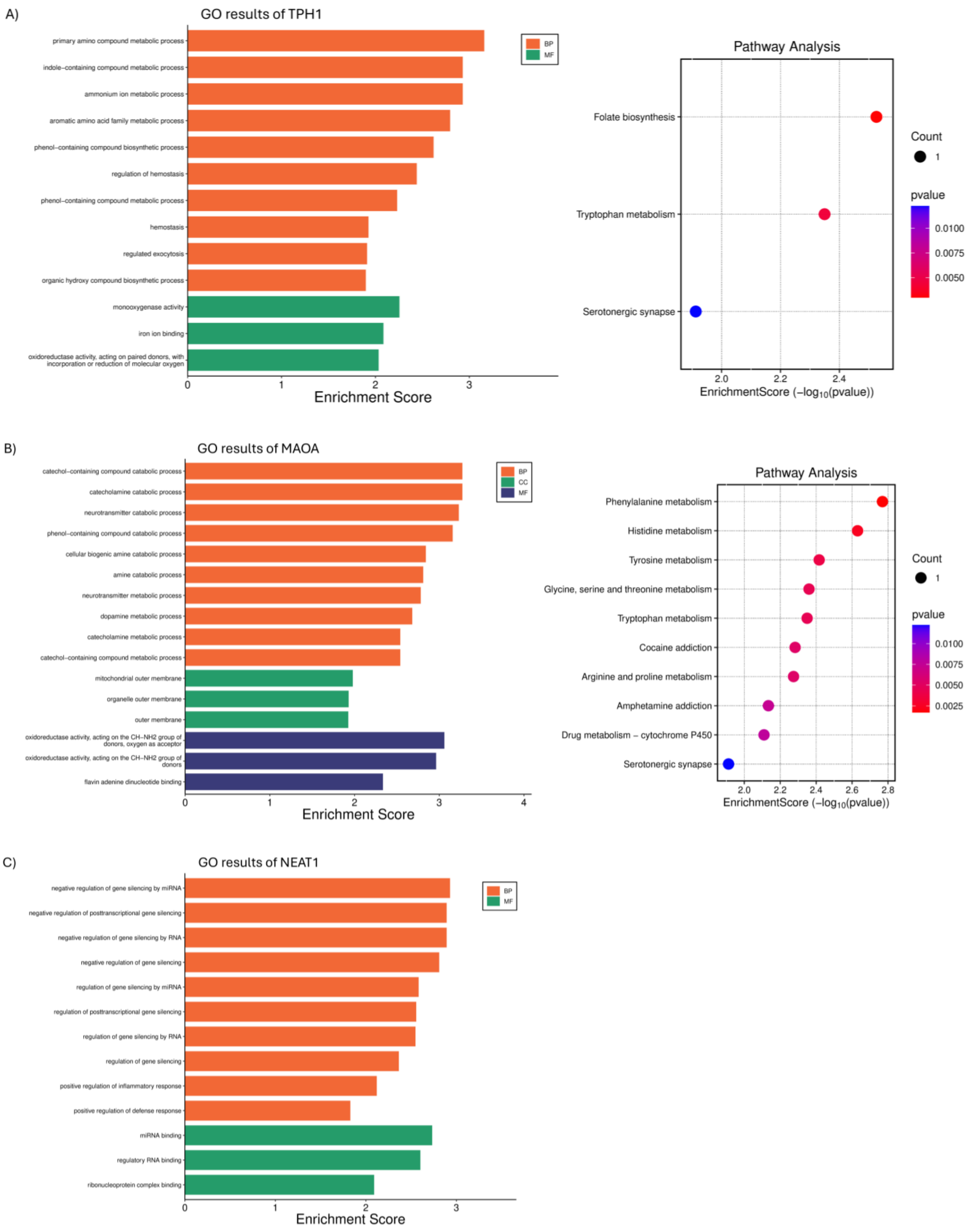
A)
GO results of TPH1
primary amino compound metabolic process
indole−containing compound metabolic process
ammonium ion metabolic process
aromatic amino acid family metabolic process
phenol−containing compound biosynthetic process
regulation of hemostasis
phenol−containing compound metabolic process
hemostasis
regulated exocytosis
organic hydroxy compound biosynthetic process
monooxygenase activity
iron ion binding
oxidoreductase activity, acting on paired donors, with incorporation or reduction of molecular oxygen
BP
MF
0
1
2
3
Enrichment Score
Pathway Analysis
Folate biosynthesis
Tryptophan metabolism
Serotonergic synapse
2.0
2.2
2.4
EnrichmentScore (−log10(pvalue))
Count
1
pvalue
0.0100
0.0075
0.0050
B)
GO results of MAOA
catechol−containing compound catabolic process
catecholamine catabolic process
neurotransmitter catabolic process
phenol−containing compound catabolic process
cellular biogenic amine catabolic process
amine catabolic process
neurotransmitter metabolic process
dopamine metabolic process
catecholamine metabolic process
catechol−containing compound metabolic process
mitochondrial outer membrane
organelle outer membrane
outer membrane
oxidoreductase activity, acting on the CH−NH2 group of donors, oxygen as acceptor
oxidoreductase activity, acting on the CH−NH2 group of donors
flavin adenine dinucleotide binding
BP
CC
MF
0
1
2
3
4
Enrichment Score
Pathway Analysis
Phenylalanine metabolism
Histidine metabolism
Tyrosine metabolism
Glycine, serine and threonine metabolism
Tryptophan metabolism
Cocaine addiction
Arginine and proline metabolism
Amphetamine addiction
Drug metabolism − cytochrome P450
Serotonergic synapse
2.0
2.2
2.4
2.6
2.8
EnrichmentScore (−log10(pvalue))
Count
1
pvalue
0.0100
0.0075
0.0050
0.0025
C)
GO results of NEAT1
negative regulation of gene silencing by miRNA
negative regulation of posttranscriptional gene silencing
negative regulation of gene silencing by RNA
negative regulation of gene silencing
regulation of gene silencing by miRNA
regulation of posttranscriptional gene silencing
regulation of gene silencing by RNA
regulation of gene silencing
positive regulation of inflammatory response
positive regulation of defense response
miRNA binding
regulatory RNA binding
ribonucleoprotein complex binding
BP
MF
0
1
2
3
Enrichment Score

Figure S3. A comprehensive functional overview of TPH1 (A), MAOA (B), and NEAT1 (C) was obtained through GO and KEGG pathway analyses.

Table S1. Primer–Probe Sequences for the Detection of mRNA, miRNA, and lncRNA by RT-qPCR.

Amplicon	RT primer F	RT primer R	Probe	Reverse	Forward
**ACTB**	-	-	Yakima Yellow-TCAAGATCATTGCTCCTCCTGAGCGC-BHQ1	GCCGATCCACACGGAGTACT	GGCACCCAGCACAATGAAG
**TPH1**	-	-	FAM-AGCCAGTCATCCAGGAACATTTGAGCAT-BHQ1	GGCCCAGACCTCCGAATT	GCAGGAAGCCGAGTATCTAACA
**MAOA**	-	-	FAM-ATGGAAGGGTGATTCGTCAACCCG-BHQ1	CACTTTGTGGCAGTCTCTGT	CTACTTCCCTCCTGGGATCAT
**NEAT1**	-	-	FAM-CTGCATCTTCTAAATTGAGCCTCCGGT-BHQ1	AGCAAGGTTCCAAGCACAA	CTTGTTCCAGAGCCCATGAAT
**hsa-miR-26b-5p**		GTCGTATGCAGTGCAGGGTCCGAGGT ATTCGCACTGCATACGACACCTAT	FAM-TG(pdC)ATA(pdC)GA(pdC)A(pdC)CTATCC-ZNA4-BHQ1	-	GCCGCTTCAAGTAATTCAGG
**hsa-miR-194-5p**	GGGATTCTGGAAGATGATG ATGACTGTAACAGCAA	GTCGTATCCAGTGCAGGGTCCGAGGT ATTCGCACTGGATACGACTCCACA	FAM-CCATGTGGAGTCGTATCCAGTGCG-BHQ1	-	GGGATTCTGGAAGATGATGATGAC TGTAACAGCAA
**hsa-miR-1276**	GGGATTCTGGAAGATGATG ATGACTAAAGAGCCCT	GTCGTATCCAGTGCAGGGTCCGAGGT ATTCGCACTGGATACGACTGTCTC	FAM-AGAGCCCTGTGGAGACAGTCGTAT-BHQ1	-	GGGATTCTGGAAGATGATGATGAC TAAAGAGCCCT
**Universal-F**	-	-	-		GGGATTCTGGAAGATGATGATGAC
**Universal-R**	-	-	-	GTGCAGGGTCCGAGGTAT	-
